# Comparison between conventional and "clinical" assessment of experimental lung fibrosis

**DOI:** 10.1186/1479-5876-6-16

**Published:** 2008-04-10

**Authors:** Kjetil Ask, Renee Labiris, Laszlo Farkas, Antje Moeller, Aaron Froese, Troy Farncombe, Grant B McClelland, Mark Inman, Jack Gauldie, Martin RJ Kolb

**Affiliations:** 1 Department of Pathology and Molecular Medicine, Center for Gene Therapeutics, McMaster University, Hamilton, Ontario, Canada; 2 Department of Medicine, Firestone Institute for Respiratory Health, McMaster University, Hamilton, Ontario, Canada; 3 Department of Nuclear Medicine, Hamilton Health Sciences, McMaster University, Hamilton, Ontario, Canada; 4 Department of Biology, Life Sciences, McMaster University, Hamilton, Ontario, Canada

## Abstract

**Background:**

Idiopathic pulmonary fibrosis (IPF) is a treatment resistant disease with poor prognosis. Numerous compounds have been demonstrated to efficiently prevent pulmonary fibrosis (PF) in animal models but only a few were successful when given to animals with established fibrosis. Major concerns of current PF models are spontaneous resolution and high variability of fibrosis, and the lack of assessment methods that can allow to monitor the effect of drugs in individual animals over time. We used a model of experimental PF in rats and compare parameters obtained in living animals with conventional assessment tools that require removal of the lungs.

**Methods:**

PF was induced in rats by adenoviral gene transfer of transforming growth factor-beta. Morphological and functional changes were assessed for up to 56 days by micro-CT, lung compliance (measured via a mechanical ventilator) and VO_2_max and compared to histomorphometry and hydroxyproline content.

**Results:**

Standard histological and collagen assessment confirmed the persistent fibrotic phenotype as described before. The histomorphological scores correlated both to radiological (r^2 ^= 0.29, p < 0.01) and functional changes (r^2 ^= 0.51, p < 0.0001). VO_2_max did not correlate with fibrosis.

**Conclusion:**

The progression of pulmonary fibrosis can be reliably assessed and followed in living animals over time using invasive, non-terminal compliance measurements and micro-CT. This approach directly translates to the management of patients with IPF and allows to monitor therapeutic effects in drug intervention studies.

## Background

Idiopathic pulmonary fibrosis (IPF) is a progressive disease of unknown origin characterized by increased matrix deposition, resulting in functional impairment and ultimately respiratory failure [1]. Despite considerable progress in understanding the pathophysiology of this disease, there is still no efficient treatment [2,3]. Animal models of pulmonary fibrosis (PF) are critical to investigate pathological mechanisms and are important for preclinical evaluation of novel therapies [4,5]. To date, several hundreds of compounds were identified in various models of experimental PF and proposed to have therapeutic potential for IPF [5]. However, only a small number have reached clinical trials and very few have been assessed in a "true" therapeutic setting [5,6], reproducing the clinical setting where patients with IPF tend to present late, with advanced fibrotic changes in their lungs [7]. The vast majority of compounds that were examined in drug intervention studies in experimental PF are only successful in a preventive or prophylactic setting, which means they are given with or prior to administration of the fibrogenic stimulus [5]. Assessment tools used in experimental PF are primarily based on histology and quantitative collagen analysis, providing a snapshot of a complex and chronic biological process [8], whereas clinical management of IPF relies on physiologic parameters (lung function, exercise test, echocardiography) and radiology (chest x-ray and high resolution computed tomography). Clinical trials are designed to detect changes in survival and/or lung function parameters [9].

We believe that intervention studies in models of PF would be more meaningful if the compound was administered in the fibrotic phase of the disease and not as prevention [8]. The purpose of this study was to characterize a late-stage non inflammatory model of experimental PF in detail [10] by comparing conventional assessment tools to clinically more applicable approaches that do not require euthanasia. The underlying idea is to establish reproducible assessment tools for experimental PF in order to apply these later in drug intervention studies. Morphological changes were quantified with histomorphometry and by analysis of 3D micro-CT images. Lung function was characterized by measuring lung elastic recoil and VO_2_max with treadmill exercise.

The data shows that micro-CT combined with invasive, non-terminal lung compliance is equally efficient as histomorphometry to generate a fibrotic grading but has the advantage that it can be done repeatedly in one animal over time. These evaluation tools directly reflect the clinical management of IPF patients. This approach is adapted to follow anatomical and functional changes in living animals over time and is well adapted to perform preclinical drug evaluation studies.

## Methods

### Animal treatment

A replication deficient adenovirus carrying active TGF-β1 (AdTGF-β1^223/225^) was used as previously described [10]. Female Sprague-Dawley rats (200–250 g) received AdTGF-β1 or control vector AdDl (1 × 10^8 ^plaque forming units/pfu) by intubation. Rats were sacrificed after day 14, 21, 35 and 56 by abdominal aorta bleeding. The animals were treated according to the guidelines of the Canadian Council of Animal Care. All animal procedures were performed under inhalation anesthesia with isofluorane (MTC Pharmaceuticals, Cambridge, Ontario) unless described otherwise.

### Histology

After fixation in 10% buffered formalin for 24 hours, lungs were paraffin embedded and stained with hematoxylin & eosin or picrosirius red. Four axial sections of the distal left lung were stained with Picro Sirius Red and 20 microscopically fields were examined by three blinded investigators. Each field was individually assessed for the severity of PF using Ashcroft's semi quantitative grading [11].

### Hydroxyproline Assay

Hydroxyproline content was determined as described previously [12]. The results were calculated as μg hydroxyproline/mg dry lung weight using hydroxyproline standards (Sigma Chemicals) and expressed as fold increase over untreated lung tissue.

### Assessment of lung stiffness: flexiVent^®^

Following anesthesia (Ketamin/Xylazine), animals were intubated via oropharynx and ventilated (10 ml/kg/breath). Pressure volume (PV) loops were measured with flexiVent^® ^(v5.1, Scireq, Montreal, PQ) allowing the rats to passively expire for 1 sec against 2 cmH_2_O positive end-expiratory pressure and then applying 7-step increases and decreases in volume of 40 ml/kg per step. The parameter K of the Salazar-Knowles equation reflects the curvature of the upper portion of the deflation PV curve and was derived from the PV-loops (v5.1). Lung stiffness was quantified by fitting the inspiratory limb of the PV loop and graphed as the volume of air necessary to reach a pulmonary pressure of 20 cm H_2_O.

### Computed tomography

Micro-CT scan of lungs were obtained using the Micro-CT-component of an X-SPECT scanner (Gamma Medica-Ideas, Northridge, California). Rats were anesthetized using inhalation isofluorane (MTC Pharma, Cambridge, ON. Canada) in a level II cabinet and inserted in a sealed Plexiglas's tube with Hepa-filters. All scans were acquired in spontaneously breathing animals over 512 angles. The total radiation dose applied to an animal using this technique is in the range of 30 mGy, which is similar to lung CT scans used in clinical medicine [13]. Reconstruction of the micro-CT images was performed at 155 μm^3 ^voxel size, converted to Hounsfield Units (HU) selecting air as -1000 and water as 0 and filtered to reduce noise (sigma = 2 voxel Gaussian 3D filter) using Amira^® ^visualization software (Mercury Computer Systems). The reconstructed image was loaded in the segmentation editor of Amira^® ^for examination. A region-growing tool was utilized to select trachea, larger airways, normal and fibrotic lung. Three-dimensional lung surfaces were generated and corresponding density histograms were extracted.

### Treadmill and VO_2 _max

Changes in gas exchange were assessed by maximal oxygen consumption VO_2_max. Mass-specific oxygen consumption (VO_2_) and CO_2 _production (VCO_2_) was measured with a flow-through respirometry system (Sable Systems, Henderson, NV) in a Plexiglas-enclosed motorized treadmill (Columbus Instruments, Columbus, Ohio). VO_2_max was measured for each animal, using three criteria: (i) no change in VO_2 _when speed is increased, (ii) rats can no longer keep their position on the treadmill, and (iii) respiratory quotient (RQ = VCO_2_/VO_2_) > 1.

### Statistical analysis

Data are shown as mean ± SEM unless stated differently. For evaluation of group differences, we used one way ANOVA with Dunnett's multiple comparison test or student's unpaired t test using GraphPad Prism^® ^5.0. P < 0.05 was considered significant.

## Results

### Conventional assessment

#### Histopathology score and collagen quantification

**Figure 1:**
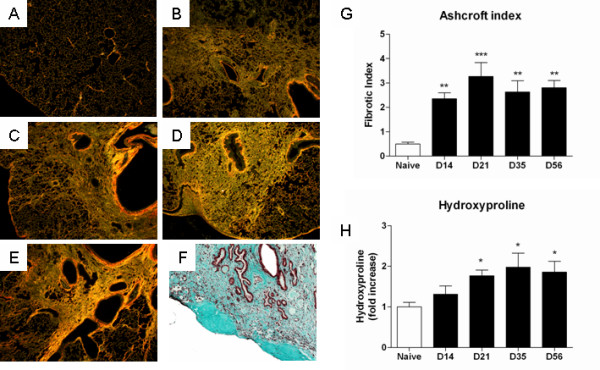
**Representative lung histology**. (A) naïve lungs, (B) day 14, (C) day 21, (D) day 35, (E) day 56 and (F) day 225 after intratracheal AdTGF-β1 administration (40×). (G) Fibrotic index (Ashcroft). (G) Hydroxyproline content per mg dry lung. Group comparison was performed using one way ANOVA with Dunnett's multiple comparison test. All values are given as mean, SE, n = 4–6 in each group, * p < 0.05, ** p < 0.01, *** p < 0.001 *vs. *naïve.

The extent of PF after transfer of active TGF-β1 was assessed histologically and biochemically and confirmed findings we reported earlier [10]. Figure 1 shows representative images of lung architecture from naïve (A) and fibrotic lungs from day 14 to 56 (B-E). Peribronchially accentuated fibrosis, not associated with inflammatory changes and loss of normal lung architecture was observed up to day 56, as well as subpleural and pleural thickening (see figure 1D). Persistent fibrosis including honeycombing was seen in later stages (up to 7 months after AdTGF-β1) (Figure 1F). Histomorphometric and hydroxyproline analysis was performed to quantify the severity of PF up to 56 days after AdTGF-β1 (Figure 1G–H).

### Clinical assessment

#### Image analysis of 3D micro-CT scans

**Figure 2:**
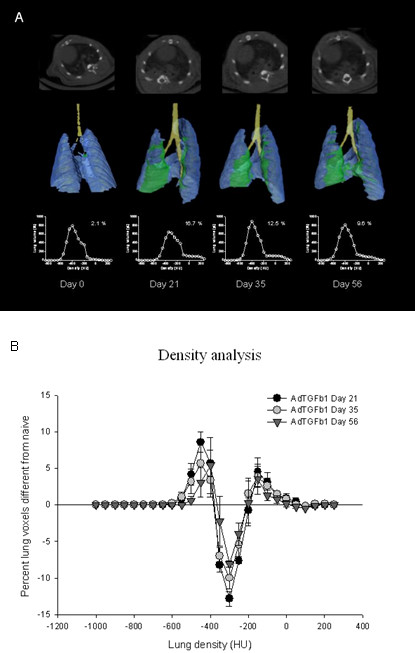
**Computed tomography**. (A). Representative example of one AdTGF-b1 exposed rat, scanned at day 0, 21, 35 and 56. Axial slice of CT-scan (upper panel) and 3D reconstructed lungs with fibrotic areas color-coded in green (medium panel). Corresponding histograms of total lung voxels from same animal (lower panel; insert represent percentage of fibrotic tissue. (B). Average percentage of voxels different from naïve lungs (All values are given as mean, SE, n = 4 to 6 at each time-point).

**Figure 3:**
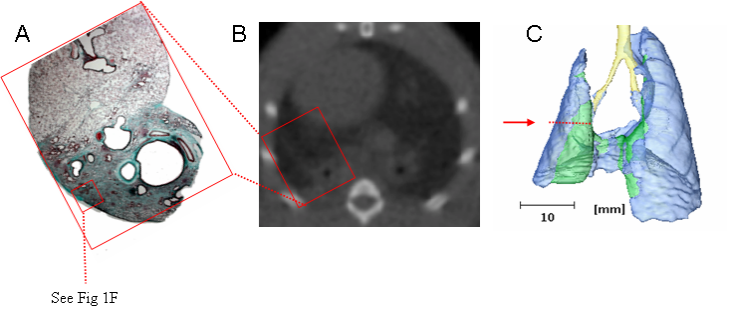
**Correlation Histology – Computed tomography**. (A). Low magnification histology 225 days after intratracheal AdTGF-β1 administration (10×) corresponding to CT image of axial slice (B) and to area indicated with red arrow in 3D reconstructed lungs from same animal (C). See insert in Fig 1F for higher magnification of same lung.

Micro-CT images were acquired over 512 angles, reconstructed at 155 μm^3 ^voxel (volume-pixel) size and converted to Hounsfield Units (HU) using a Matlab program where -1000 is defined as the density of air and 0 the density of water. Axial slices of micro-CT scans of fibrotic lungs at day 21, 35 and 56 showed areas of denser tissues compared to naïve lungs (Figure 2A, upper panel), correlating to fibrotic areas in histology slides. Three-dimensional regions were selected and color-coded as follows: trachea (yellow), normal lung density (blue, -600 to -100 HU), and high lung density (green, -100 to 200 HU). This allowed visualization of lung areas with different densities (Figure 2A, medium panel) and quantification after generating corresponding histograms (Figure 2A, lower panel). A shift to the right (= denser tissue) was observed from (see insert) day 21 to 56. Histograms of fibrotic rats (n = 6) at each time point were selected as described above and the number of voxels in each bin was subtracted from a group of unexposed animals. Figure 2B shows the differential increase and/or decrease of specific voxel ranges compared to naïve lungs: increase of high dense voxels corresponding to fibrotic regions (ranging from-100 to 250 HU), and increase of lower density voxels (ranging from-600 to -400 HU), possibly reflecting airspace enlargement (e.g. compensatory mechanism or traction effect) This increase is accompanied by a decrease of normal density voxels (-200 to -400 HU). A lower magnification histology and CT image is provided in Figure 3 which indicates that this model is persisting up to 225 days after intratracheal AdTGF-β1 (see Fig 1F for higher magnification)

#### Lung function and VO_2_max

**Figure 4:**
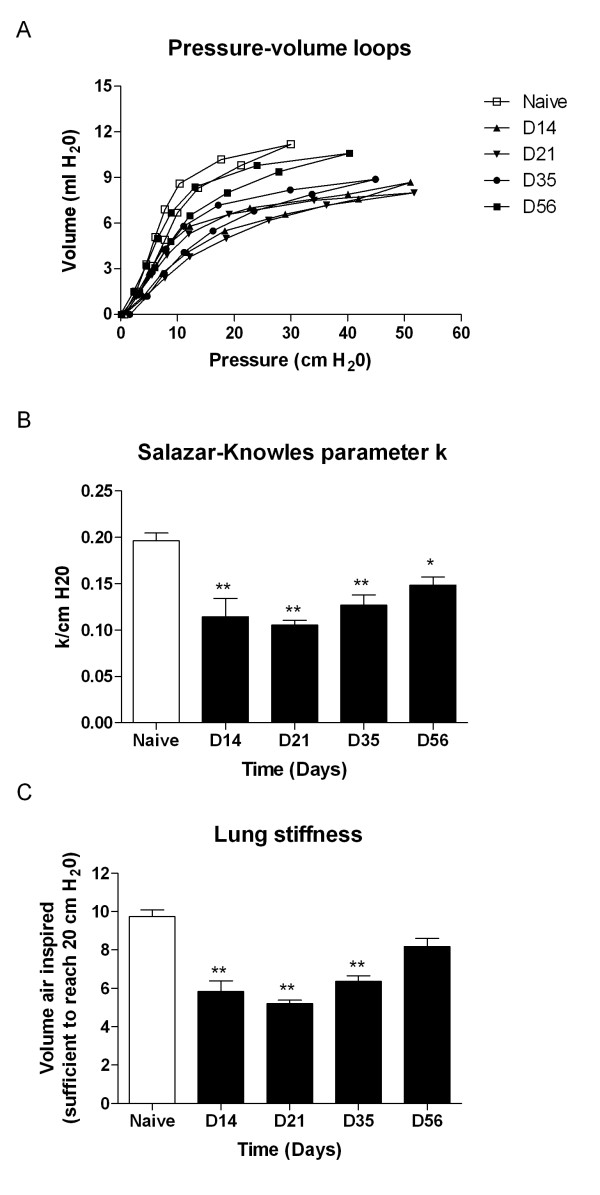
**Lung function**. (A) Average pressure/Volume curves from naïve and AdTGF-β1 exposed rats. The curves from fibrotic animals demonstrate marked shifts downward and to the right, indicating stiffer lungs. Note: The upward deviation of the curve at day 14 at pressures above 60 cm H2O reflect the upper limits of the pressure transducer. (B) The parameter K was reduced at all time-points. (C) Lung stiffness was derived from the PV loop in Figure 4A and characterized as the volume of air needed to reach a pressure of 20 cm H_2_O. All values are given as mean, SE, n = 4–6 per group.

Rats were intubated via oropharynx and two pressure-volume (PV) loops were performed for each animal using flexiVent^®^. Figure 4A shows the average of all PV-loops at different time-points. The factor K of the Salazar-Knowles equation [14] is presented in Figure 4B and demonstrate a significant decrease which persisted over at least 56 days. The day before the end of the experiment, rats were placed on a closed treadmill to run at increasing speeds while oxygen and C0_2 _were measured continuously. VO_2 _max was reduced on average by 10% but this did not reach statistical significance (data not shown).

### Correlation between conventional and clinical assessment

**Figure 5:**
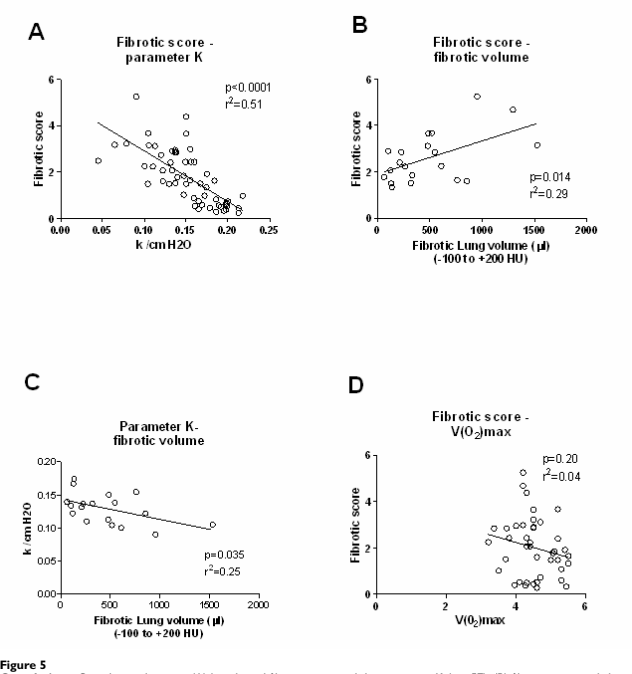
**Correlations**. Correlations between (A) histological fibrotic score and the parameter K (n = 57), (B) fibrotic score and absolute fibrotic volume (n = 20), (C) parameter K and absolute fibrotic volume (n = 18) and (D) fibrotic score and VO_2_max (n = 41).

The histomorphology score (Ashcroft) was compared to lung function, and showed an excellent correlation to the parameter k (Fig 5A, p < 0.0001). The fibrotic volumes in micro-CT, defined as high dense voxels comprised between -100 to +200 HU, correlated both with the fibrotic score (p = 0.014, Figure 5B) and to the parameter k (p = 0.035, Figure 5C). There was no correlation to the decrease in VO_2 _max (Figure 5D).

## Discussion

The purpose of this project was to evaluate non-terminal, "clinical" assessment tools in an animal model of experimental PF and compare them with standard methods used in the field. The obvious advantages of non-terminal tools are that severity and progression of fibrotic changes or – in the context of drug intervention studies – their potential reversibility can be followed in individual animals over time. This reduces variability and the number of animals that are needed to create relevant time-course data. In this study, we used an established model of PF which is based on adenovirus mediated overexpression of active TGF-β1 in rat lungs [10]. This model incorporates early fibroproliferation while the transgenic protein is still expressed, not associated with significant inflammation (up to 10 days), followed by progressive and persistent fibrosis, which is driven by endogenous processes, likely mediated by locally activated TGF-β [10,15].

Current assessment tools for animal models of PF are based on histology and biochemical analysis of lung tissue [16,17]. It is common practise in experimental models to use one lung for histology while the other lung is frozen and homogenized for biochemical and/or molecular analysis. Histomorphometry is considered as the gold standard. The method of Ashcroft requires multiple photomicrographs randomly acquired across the lung parenchyma and subsequent scoring by several investigators ranging from 0 to 8, where 8 would be a completely obliterated fibrotic field [11]. We adapted this approach in the present study with blinded scoring of tissue samples by three different investigators. This method produces solid and reproducible data, but it is time consuming and only a small number of published studies seem to rely on it. Further, the scoring is usually performed on 20 high-power magnification images, which only accounts for a small fraction of the total lung, containing the risk of selection bias.

The quantification of collagen, or its major amino acid hydroxyproline, is extensively used in PF models as readout for disease severity and success of antifibrotic therapy. Compounds are considered unsuitable for fibrosis therapy if they have not shown a substantial decrease in lung hydroxyproline content in an appropriate model. There are a number of methodological issues associated with collagen quantification in the lungs. In experimental PF, the amount of hydroxyproline rarely exceeds a two-fold increase over baseline, and the levels are highly variable [18], narrowing the therapeutic window. This may partly explain why most published studies use a "prophylactic setting", giving the drug prior to initiation of fibrosis (usually by intratracheal bleomycin) [5,19]. With this experimental design it can be assumed that hydroxyproline levels in drug-treated animals were never increased to a similar extend as in control animals. This means, the tested compound interferes with a process that is biologically different from the one in established fibrosis. Further, there is a difference between content and concentration of lung collagen. In our study we have quantified hydroxyproline related to dry lung weight to correct for potential pulmonary fluid accumulation or edema. Some determine collagen per gram wet weight, while others present total content per lung independent of the weight. Finally, it is likely that ultra-structural and qualitative changes in fibrotic matrix, such as collagen fiber stiffness or fiber cross-links markedly impact lung elasticity [20]. These changes cannot be determined by quantitative analysis of hydroxyproline or histomorphometry, but may be captured by physiological measurements.

The routine assessment of IPF patients in clinic includes measurement of lung volumes, usually forced vital capacity (FVC) and total lung capacity (TLC). These parameters are difficult to obtain in rodents. In contrast, compliance can easily be measured and it has been shown that this parameter of lung stiffness is reduced in IPF patients and correlates with disease severity [21]. We systematically performed pressure volume loops in live rats using an automated ventilator to assess changes in lung elasticity. The parameter K of the Salazar-Knowles equation, a reflection of the curvature of the upper portion of the pressure-volume curve, was reduced by 35 % in animals with PF up to 56 days after AdTGF-β1. There was a strong correlation between this "stiffness" parameter and the fibrosis score. The major advantage of this method is that it can be performed repeatedly in the same animals. We have here used a rat model of PF, but this method is applicable to both mice and rats [22-24].

In addition to lung function, human clinical trials are powered to detect differences in exercise tests, mostly the 6 minute walk [25]. Therefore, we have investigated the usefulness of VO_2_max employing a rodent treadmill system in a contained Plexiglas tube. VO_2_max was on average reduced in fibrotic rats by 10% compared to controls, but did not allow us to clearly distinguish between the two groups. It is still unclear why VO_2_max alterations were less than expected. The total fibrotic volume defined by CT scan analysis accounted for 15–20% and it is possible that this is not sufficient to reduce VO_2_max significantly. It may also be that compensatory lung growth and recruitment of alveolar reserves occurs in this rodent model, thus preventing the progressive functional decline that is so characteristic for lung fibrosis in humans. Further studies are required to better understand the complex changes in cardiopulmonary physiology in animal models of lung fibrosis. However, the presented data do not support VO_2_max as a useful tool for assessment of fibrosis severity in this model.

Radiologic examination of lungs including high resolution computed tomography is a mandatory tool to make a clinical diagnosis of IPF/UIP [1]. When CT scans are post-processed, three dimensional structures can be displayed with density histograms, making it an ideal candidate to follow total lung parenchymal changes over time. This approach is more widely propagated for chronic disorders characterized by tissue loss such as emphysema [13,26,27], but may be of significant value in fibrotic lung disease. Several software packages are commercially available, allowing extraction of tissue areas of choice which are defined by certain pre-set parameters. After 3-D reconstruction, these areas can be measured and the volume of lung tissue characterized by a specific density range can be quantified. In CT images, fibrotic lung tissue appears denser compared to normal lungs. In contrast, a shift towards lower densities would be associated with air-space enlargement, emphysema or increase in lung volume. While it is relatively easy to "virtually dissect" areas with fibrotic density from normal lung, it is more difficult to clearly distinguish between fibrosis and mediastinal structures such as heart, aorta and vena cava. To circumvent this, we use a semi-automated 3D extraction of lung tissues with help of a "segmentation editor" and a "region growing tool". The volume of fibrotic tissue in our study was in the range of 10–20% of total lung and correlated significantly to conventional fibrosis score and lung function indicating that micro-CT is a promising alternative to predict lung fibrosis as previously described in the bleomycin model using mice [28] or rats [29]. It is important to note that this CT technique does not distinguish between inflammatory and fibrotic changes in the lung tissue. Other imaging such as PET [30] or MRI [31] technology might be better suitable to differentiate between inflammation, edema and fibrosis, which is a relevant consideration when inflammatory models of fibrosis are used (e.g. the bleomycin model, in contrast to our non-inflammatory AdTGF-β1 model).

The major benefit of micro-CT combined with lung compliance over the more traditional tools histology and collagen content is that these assessments can be done repeatedly throughout the course of an experiment. It is obvious that validation experiments need to be performed to demonstrate that these assessments do not interfere with the progression of experimental disease. Our approach to future drug efficacy studies with these novel tools would be to obtain baseline evaluation in each animal before fibrosis is induced, then confirmation of fibrosis before the treatment is initiated and subsequent evaluation of potential treatment effects. For this purpose, a minimum number of 3 to 5 animals per group will be required, depending on the severity of fibrosis and the magnitude of treatment effects [8]. The overall aim of the new approach is to directly track if documented fibrotic changes at one point of the study can be reversed by the drug intervention at a later time point.

Further, this approach assures that the entire lung is assessed in each animal, while the conventional tools are limited by performing histology on one and biochemical analysis on the other side of the lungs. While we believe that this novel approach eventually will prove to be superior compared to terminal assessment in detecting beneficial drug effects, we acknowledge that a series of drug intervention studies needs to be done to underscore this hypothesis.

## Conclusion

In conclusion, we have translated clinically relevant assessment tools into an animal model of severe lung fibrosis and compared them with conventional methods. We show that the extent of fibrosis can be reliably monitored with small animal CT imaging and lung compliance measured with a rodent ventilator. These measurements have the advantage that they can be performed repeatedly in the same animals over time.

## Competing interests

The author(s) declare that they have no competing interests.

## Authors' contributions

KA conceived and coordinated the study, carried out the animal work, data analysis and wrote the draft of the paper. RL conceived and coordinated the study, carried out the animal work, data analysis and wrote the draft of the paper. LF participated in the design of the study, helped with the animal work and carried out the histological work. AM helped to carry out the animal work, histology analysis and edit the manuscript. AF helped in the design of the study, and CT analysis of the study. TF helped in the analysis and management of the computed tomography data. GM performed the VO2max analysis and helped in the analysis of the data. MI helped in the design of the study and in the analysis of the pressure volume loops. JG helped to conceive the study, participated in the design of the study and edited the paper. MK helped to conceive the study, participated in the design of the study, provided general supervision and helped to draft and edit the paper. All authors read and approved the final manuscript.
